# Understanding functional and social risk characteristics of frail older adults: a cross-sectional survey study

**DOI:** 10.1186/s12875-018-0851-1

**Published:** 2018-10-19

**Authors:** David R Lee, Eilann C Santo, Joan C Lo, Miranda L Ritterman Weintraub, Mary Patton, Nancy P Gordon

**Affiliations:** 1 Department of Medicine, Kaiser Permanente Oakland Medical Center, 3600 Broadway, Oakland, CA 94611 USA; 2 Graduate Medical Education, Kaiser Permanente Oakland Medical Center, 3600 Broadway, Oakland, CA 94611 USA; 3 Division of Research, Kaiser Permanente Northern California, 2000 Broadway, Oakland, CA 94612 USA

**Keywords:** Frailty, Falls, Frailty index, Social determinants, Modifiable health risks, Older adults, Primary care

## Abstract

**Background:**

Frailty is a condition of increasing importance, given the aging adult population. With an anticipated shortage of geriatricians, primary care physicians will increasingly need to manage care for frail adults with complex functional risks and social-economic circumstances.

**Methods:**

We used cross-sectional data from 4551 adults ages 65–90 who responded to the 2014/2015 cycle of the Kaiser Permanente Northern California Member Health Survey (MHS), a self-administered survey that covers multiple health and social characteristics, to create a deficits accumulation model frailty index, classify respondents as frail or non-frail, and then compare prevalence of functional health issues including Activities of Daily Living (ADL)/Instrumental Activities of Daily Living (IADL) and social determinants of health (SDOHs) by frailty status.

**Results:**

The overall prevalence of frailty was 14.3%, higher for women than men, increased with age, and more common among those with low levels of education and income. Frail older adults were more likely than non-frail to have ≥ 3 chronic diseases (55.9% vs. 10.1%), obesity (32.7% vs. 22.8%), insomnia (36.4% vs. 8.8%), oral health problems (25.1% vs. 4.7%), balance or walking problems (54.2% vs. 4.9%), ≥ 1 fall (56.1% vs. 19.7%), to use ≥ 1 medication known to increase fall risk (56.7% vs. 26.0%), and to need help with ≥2 ADLs (15.8% vs. 0.8%) and ≥ 2 IADLs (38.4% vs. 0.8%). They were more likely to feel financial strain (26.9% vs. 12.6%) and to use less medication than prescribed (7.4% vs. 3.6%), less medical care than needed (8.3% vs 3.7%), and eat less produce (9.5% vs. 3.2%) due to cost. Nearly 20% of frail adults were unpaid caregivers for an adult with frailty, serious illness or disability.

**Conclusions:**

This study examined the prevalence of frailty and identified modifiable and non-modifiable risk factors of health. The frail older adult population is heterogeneous and requires a patient-centered assessment of their circumstances by healthcare providers and caregivers to improve their quality of life, avoid adverse health events, and slow physical and mental decline. The characteristics identified in this study can be proactively used for the assessment of patient health, quality of life, and frailty prevention.

## Background

Adults aged 65 and older are expected to comprise 21.7% of the U.S. population in 2040, up from 14.5% in 2014 [1]. With the exponential growth of the aging population, frailty has become a syndrome of increasing public health concern due to the projected impact on utilization of healthcare resources and age-related services [2]. Frailty in older adults is a clinical syndrome described as a loss of “physiologic reserve” from an accumulation of physical, environmental and psychosocial factors [3–5]. These factors are often referred to as deficits that increase vulnerability to undesirable health outcomes [6].

There is currently no clear consensus on how to define and assess frailty. Some studies have defined frailty by using phenotypic characteristics such as weakness, unintentional weight loss, slow walk speed, exhaustion, and low physical activity [4], whereas other studies have identified a series of health-related deficits to calculate a Frailty Index (FI) and determine degree of frailty [7, 8]. To qualify as a deficit, the prevalence of the deficit must increase but not become universal with age and must include more than one organ system [8, 9]. Despite the differences in the operationalization of frailty, increasing age, female sex, lower educational attainment levels, and adverse health outcomes including falls, disability, hospitalizations, and mortality have been consistently associated with frailty in older adults [4, 6, 10].

Frailty can also be conceived as a dynamic process in which older adults transition between different states of frailty over time [11]. By screening for modifiable risk factors, interventions can potentially alter frailty status, prevent adverse health outcomes, and improve quality of life [12]. Recognizing these modifiable and even non-modifiable risk factors among community-dwelling frail older adults continues to remain a topic of interest and study.

Research suggests that approximately 30% of the 65 and older patient population have health problems complex enough to require care by a geriatrician [13]. However, a recent report by the Health Services Resource Administration in the U.S. Department of Health and Human Services projects that, based on this need, there will be a national shortage of nearly 27,000 geriatricians in 2025 [14]. The implication of this shortage is that adult primary care clinicians will increasingly be called on to manage care for older adults with complex needs such as frailty, with fewer geriatricians available for consultation. Effective care of this population will require the development of geriatric health risk assessment tools and care management protocols to allow primary care clinicians to more effectively identify and intervene on social-economic and health issues in ways that improve quality of life and prevent adverse health outcomes.

In this study, we attempt to identify potentially modifiable health and social-economic risks in the older adult patient population which, if appropriately addressed, may reduce or slow the pace of health and functional decline and improve quality of life among those with frailty or complex needs. This study aims to encourage further assessments and interventions in the broader older adult population that might help reduce the prevalence of frailty in the aging population.

## Methods

### Study cohort and data source

**Fig. 1:**
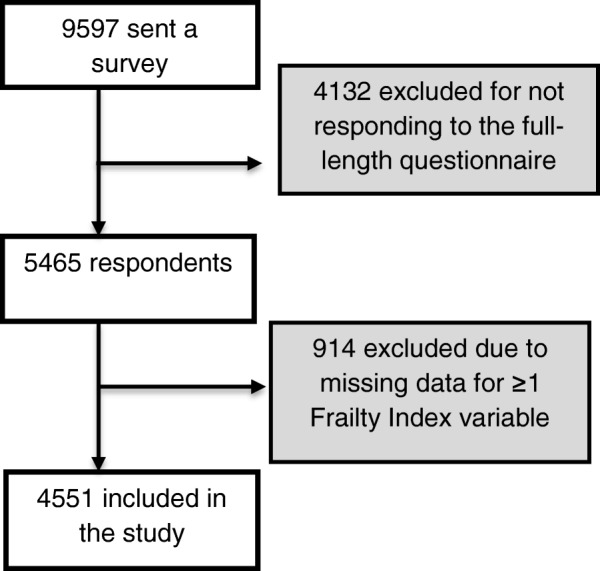
Flow diagram of included and excluded participants

This cross-sectional observational study used self-reported data for respondents aged 65–90 from the 2014/2015 cycle of the Kaiser Permanente Northern California (KPNC) adult Member Health Survey (MHS). The MHS is a self-administered (mailed and online) general health survey conducted with stratified random samples of English-speaking KPNC members aged 20 and over, with oversampling of adults aged ≥ 65 [15]. The MHS is representative of the overall KPNC health plan membership in terms of demographic characteristics and reflective of the insured adult population in Northern California [15]. The survey covers sociodemographic characteristics, social determinants of health (SDOH), health and functional status, health-related lifestyle behaviors, and psychosocial risks. Figure 1 includes a diagram of included and excluded participants. In the 2014/2015 cycle, the response rate for ages 65–90 was 56.9% (5465/9597) for the first two mailings which included the full-length questionnaire. We further restricted the study sample to respondents who were not missing data for any of the items used in the FI (4551/5465), resulting in a study cohort that was 47.4% of the original sample of 9597 who were sent the mailed or online survey. A comparison of the final study sample, after weighting to the population, found that the study cohort was very similar to the full respondent sample (which included respondents who completed a shorter version of the questionnaire used in the final third mailing) with regard to age, sex, race/ethnicity, education, income, self-reported health, and frequency that health problems interfered with daily activities. KPNC’s Institutional Review Board approved the MHS and use of MHS data for descriptive studies.

### Sociodemographic characteristics

Sociodemographic factors examined in this study included age, sex, race/ethnicity, educational attainment, relationship status, and household income. Age was grouped into categories: 65–69, 70–74, 75–79, 80–84, and 85–90 years. Race/ethnicity was categorized as non-Hispanic white, African-American/black, Hispanic/Latino, Asian/Pacific Islander, and other. Educational attainment level was categorized as less than high school graduate, high school graduate or equivalent, some college or associates degree, or college graduate. Relationship status was categorized as married or in a committed relationship, widowed, or single/divorced/separated. Household income was separated into less than $25,000, $25,001–$35,000, $35,001–$50,000, $50,001–$65,000, $65,001–$80,000, and greater than $80,000. Approximately 8% of respondents in the study group were missing data for income.

### Frailty index components

For each respondent, we created a Frailty Index (FI) score based on a 34-point accumulation of deficits model [6, 7]. The deficits chosen for this study were similar to deficits used in prior indices [6–9]. Similar to other studies, our study is a secondary analysis of an already existing dataset and therefore, used deficits from our questionnaire that were similar to those used to create prior frailty indices from the Canadian Study of Health and Aging [6], the Yale Precipitating Events Project [8], and the National Population Health Survey of Canada [9]. Deficits were based on prior FI guidelines by Searle et al. [8]: they were biologically sensible, increased but did not become universal too early with age, and ranged across a variety of organ systems [8, 9]. These deficits were all based on self-reported data.

Table 1 summarizes the health and functional status variables used to assign FI points. Eight categories drawn from self-reported MHS survey responses were used to create a frailty point score. These included: self-rated overall health, extent to which physical and emotional health interfered with activities of daily living, history of 9 selected chronic diseases ascertainable from the survey, 9 chronic functional health conditions, mobility problems, 11 activities of daily living/instrumental activities of daily living (ADLs/IADLs), and underweight based on a body mass index (BMI) of < 18.5 kg/m^2^ calculated using self-reported height and weight information. A maximum of 1 point was assigned for each health-related deficit, for a maximum total of 34 points. The FI was calculated by dividing the frailty point score by 34. An individual was classified as frail if he/she had a FI score of 0.2 or above as described in a prior study by Song et al. [8].

**Table 1:** List of deficits and point criteria used to calculate frailty index

Point Criteria		Maximum 34 Points
In general, would you say your health is: ▪ Excellent = 0 ▪ Very Good = 0.25 ▪ Good = 0.5 ▪ Fair = 0.75 ▪ Poor = 1		1 point
How much does your physical health interfere with your work or other regular daily activities? ▪ Not at all = 0 ▪ A Little Bit = 0.25 ▪ Moderately = 0.5 ▪ Quite a bit = 1		1 Point
How much does your emotional/mental health interfere with your work or other regular daily activities? ▪ Not at all = 0 ▪ A Little Bit = 0.25 ▪ Moderately = 0.5 ▪ Quite a bit = 1		1 point
Chronic diseases = 1 point for each		9 points
▪ Diabetes ▪ Hypertension ▪ History of Heart disease ▪ History of Cancer (other than just skin) ▪ Osteoarthritis	▪ Osteoporosis ▪ History of Stroke ▪ Parkinson’s Disease ▪ COPD/Bronchitis	
Chronic functional health conditions = 1 point for each		9 points
▪ Vision problems that interfered with daily activities (driving, reading, etc.) ▪ Hearing problems and/or deafness ▪ Urinary leakage ▪ Depression/Anxiety ▪ Frequent/ongoing Pain	▪ Oral health problems making it difficult to eat or talk ▪ Balance or walking problems ▪ Memory problems ▪ Insomnia	
Mobility problems: Which of the following describes your situation? ▪ I usually need help from another person to move around or I usually use a motorized wheel chair = 1 ▪ I usually use a cane, walker or poles = 0.5 ▪ I don’t need help, but have some trouble getting around = 0.25 ▪ I am not limited at all in my ability to get around = 0		1 point
Help needed with ADLs or IADLs due to a disability, health problem, or frailty due to age = 1 point for each		11 points
ADLs: ▪ Getting in and out of bed/chairs ▪ Bathing in a tub or shower ▪ Dressing ▪ Eating food/drinking liquids ▪ Using the toilet	IADLs: ▪ Shopping for groceries ▪ Doing laundry/household chores ▪ Preparing meals ▪ Managing money ▪ Managing/taking your medicines ▪ Using the telephone	
Underweight based on Body Mass Index (BMI, kg/m^2^) ▪ BMI < 18.5 = 1 BMI ≥ 18.5 = 0		1 point

### Additional risk characteristic variables

In addition to the variables used to create the FI, we examined several health-related behaviors, lifestyle, and psychosocial risks, as well as factors identified by the Institute of Medicine as SDOH [16]. The health-related risks, all of which are potentially subject to intervention, include number of hours of sleep per day (≤ 5 and > 9), number of falls in the prior 12 months (≥ 1 and ≥ 2), regular (≥ 2 times a week) use of a medication that can increase risk of falling (prescription anti-depressant, anti-anxiety, and/or pain medicine and prescription or over-the-counter sleep medicine), individual’s perception of his/her ability to take care of him/herself, obesity, low exercise frequency, and no dental examination or teeth cleaning within the past 12 months. The SDOH risk factors which may or not be modifiable but are important to consider as part of care management planning include low educational attainment (≤ high school graduate or equivalent), low household income based on regional data (≤ $25,000 and ≤ $35,000), relationship status (married or in a committed relationship vs. single or widowed), financial strain and markers for financial strain (worried a great deal about financial situation in past year; reduced use of medical care due to cost in past year; reduced use of prescription medications due to cost in past year; reduced fruit/vegetable consumption due to cost in past year), frequent loneliness/social isolation, frequent depression/sadness, dissatisfaction with life, inability to use the internet or email without help, and serving as an unpaid caregiver to another adult who is frail or has a serious illness or physical/mental disability.

### Statistical analysis

All analyses were performed using survey data weighted to reflect the underlying age-sex and geographic distribution of adults in the KPNC membership in 2014. We used PC-SAS version 9.3 (SAS Institute, Inc., Cary, NC) procedures for analyzing data obtained from complex survey designs [17]. Prevalence estimates are reported with 95% confidence intervals (CI) or standard errors (SE) and the chi-square test was used to examine differences by subgroup or patient characteristic. A two-tailed *p*-value criterion of < 0.05 was chosen as the threshold for statistical significance. We did not adjust for multiple comparisons but report the results of all statistical tests performed.

## Results

### Characteristics of the weighted study cohort

There were 4551 participants included in this study. The average age of the study sample was 73.3 years, with 37.8% aged 65–69, 24.5% aged 70–74, 17.2% aged 75–79, 11.9% aged 80–84, and 8.6% aged 85–90. Females made up 54.5% of the cohort sample, and 74.2% were non-Hispanic white, 5.4% African-American/other black, 7.0% Hispanic/Latino, 11.2% Asian/Pacific Islander, and 2.2% other race/ethnicity. The cohort was fairly well-educated, with 4.3% not having graduated from high school, 20.4% having a high school diploma, 33.1% having attended some college or earning an Associate’s degree, and 42.2% having a baccalaureate or postgraduate degree. In terms of relationship status, 66.3% were married or in a committed relationship, 16.6% were widowed, and 17.1% were single, divorced, or separated. Approximately 26% had a household income (HHI) of ≤ $35,000 (including 14.8% ≤ $25,000), 27.4% a HHI between $35,000–$65,000, 12.3% a HHI between $65,000–$80,000, and 33.7% a HHI > $80,000. Nearly 45% rated their health as excellent or very good, 39.1% as good, 13.1% as fair, and 2.5% as poor.

### Prevalence of frailty by demographic characteristics

**Fig. 2:**
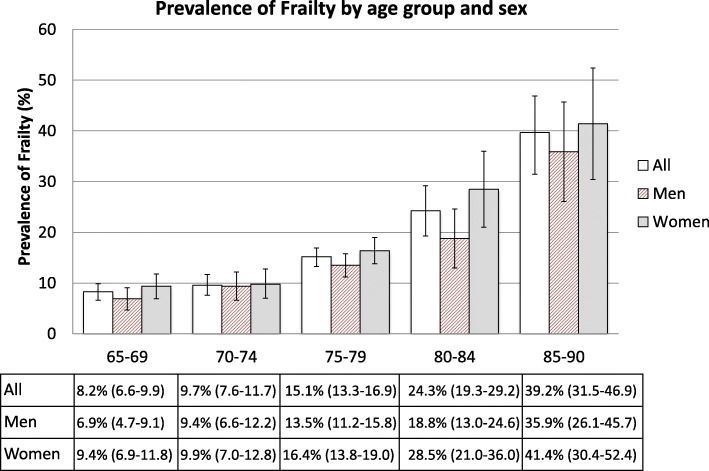
Prevalence of frailty by age group and sex

The overall prevalence of frailty (defined by FI ≥ 0.2) was 14.3% (CI: 13.0–15.6%), and was significantly higher for women (16.1%, CI: 14.1–18.1%) than men (12.2%, CI: 10.6–13.8%). Figure 2 shows that while the prevalence of frailty in the 65–69 and 70–74 age groups was not significantly different (8.2% and 9.7%, respectively, *p* > .05), frailty increased significantly for each subsequent 5-year age category (15.1% for ages 75–79, 24.3% for ages 80–84, and 39.2% for ages 85–90, *p* < .05). In addition, when stratified by 5-year age groups, frailty prevalence did not significantly differ by sex except for the 80–84 age group. Table 2 shows that while frailty status did not differ by race/ethnicity, it was significantly more common among those with low levels of education and income (e.g., 33.4% of non-high school graduates, *p* < .0001; 32.4% of those with HHI ≤ $25,000, *p* < .0001). Prevalence of frailty was also significantly higher among adults who were not currently married or in a committed relationship.

**Table 2:** Prevalence of frailty^a^ among adults aged 65–90, by sociodemographic characteristics

	Unwtd. N	Wtd. %	% Frail (95% CI)	*p*-value
Race/Ethnicity,%				ns
White non-Hispanic	3293	72.3	14.2 (12.7–15.8)	
African-American/Other black	262	5.8	16.7 (11.3–22.1)	
Hispanic/Latino	362	7.9	17.4 (12.5–22.1)	
Asian/Pacific Islander	540	11.9	12.5 (9.1–15.9)	
Other	94	2.1	11.6 (5.1–18.0)	
Education,%				*p* < .0001
< High school graduate	217	4.8	33.4 (25.2–41.6)	
High school graduate or equivalent	987	21.8	18.6 (15.4–21.8)	
Some college or associates degree	1484	32.8	13.6 (11.4–15.8)	
College graduate	1836	40.6	10.8 (9.0–12.6)	
Relationship Status,%				*p* < .0001
Married or in a committed relationship	3020	67.0	11.5 (10.1–12.9)	
Single, widowed, divorced, or separated	778	17.3	19.7 (17.1–22.4)	
Household income^b^,%				*p* < .0001
< $25,000	649	15.7	32.4 (27.5–37.3)	
$25,001–$35,000	491	11.8	21.5 (17.0–25.9)	
$35,001–$50,000	660	15.9	13.2 (10.1–16.4)	
$50,001–$65,000	511	12.3	11.6 (7.9–15.2)	
$65,001–$80,000	521	12.6	9.2 (6.2–12.2)	
> $80,000	1315	31.7	7.0 (5.4–8.7)	

Estimates are based on self-reported survey data weighted to the age-sex composition of KPNC seniors in 2014

^a^Seniors were considered frail if the average of their frailty point score was ≥0.2 based on dividing total frailty points by the 34 point maximum

^b^Approximately 8% of seniors were missing data on household income

### Frailty and functional health issues

Table 3 shows health and functional issues overall for the senior cohort and by frailty status. As might be expected based on how the FI was created, the frail group was significantly more likely than the non-frail group to report fair or poor health (55.0% vs. 9.0%, p < .0001) and poor health (14.6% vs. 0.5%, *p* < .0001) and to report ≥3 of the chronic diseases included in the FI (55.9% vs. 10.1%, *p* < .0001). Two-thirds of the frail group experienced frequent or ongoing pain, and 80.1% indicated that physical health problems interfered at least moderately with their daily activities. A significantly higher proportion of the frail compared to non-frail group had issues with sleep, including problems falling or staying asleep and getting ≤5 h or > 9 h of sleep per day. Approximately half of the frail group experienced urinary leakage more than once per week, with this symptom being more common among women than men (59.0% vs. 32.9%, *p* < .0001).

**Table 3:** Health and functional status characteristics of seniors, by frailty status

	All	Frailty Status		
	(*N* = 4551) Wtd.%	Frail^a^ (*N* = 647) Wtd. %	Non-Frail (*N* = 3904) Wtd. %	*p*-value Frail vs. Non-Frail
Self-reported overall rating of health				
Very good/excellent	45.3	7.2	51.7	*p* < .0001
Good	39.1	37.9	39.3	
Fair	13.1	40.4	8.5	
Poor	2.5	14.6	0.5	*p* < .0001
Has physical health problems that interfere at least moderately with daily activities^b^	27.6	80.1	18.8	*p* < .0001
Has emotional/mental health problems that interfere at least moderately with daily activities^b^	11.2	38.3	6.7	*p* < .0001
Has ≥3 chronic diseases^b,c^	16.7	55.9	10.1	*p* < .0001
Has headaches, migraine, musculoskeletal pain, or other frequent/ongoing pain^b^	30.5	65.6	24.6	*p* < .0001
Often feels depressed or is being treated for depression^b^	10.3	29.2	8.4	*p* < .0001
Has problems falling or staying asleep^b^	12.7	36.4	8.8	*p* < .0001
Usually gets > 9 h of sleep per day	7.0	14.7	5.7	*p* < .0001
Usually gets ≤5 h of sleep per day	5.7	10.8	4.9	*p* < .0001
Has oral health problems that affect eating or speech^b^	7.6	25.1	4.7	*p* < .0001
Has vision problems that affect daily activities^b^	9.9	27.4	7.0	*p* < .0001
Has hearing problem^b^	24.4	50.8	19.9	*p* < .0001
Experiences urine leakage ≥ once/week^b^	19.4	48.9	14.5	*p* < .0001
Has frequent problems with balance or walking	12.0	54.2	4.9	*p* < .0001
Has mobility limitations^b^				
None	78.6	24.6	87.6	*p* < .0001
Some difficulty moving around	10.4	24.6	8.0	
Uses a cane or poles when moving around	8.5	35.0	4.0	
Uses a motorized device or needs help from a person to get around	2.5	15.7	0.4	*p* < .0001
Had ≥1 fall in prior 12 months	24.7	56.1	19.7	*p* < .0001
≥ 2 falls in prior 12 months	11.3	33.3	7.7	*p* < .0001
Uses medication ≥2 times/week that can increase risk of falling				
Prescription anti-depressant	7.8	20.0	5.8	*p* < .0001
Prescription anti-anxiety medication	4.8	15.1	3.1	*p* < .0001
Prescription pain medication	17.6	42.4	13.4	*p* < .0001
Prescription or over-the-counter sleep medicine	12.1	20.4	10.7	*p* < .0001
≥ 1 of above types of medication	30.4	56.7	26.0	*p* < .0001
≥ 2 of above types of medication	9.5	29.7	6.1	*p* < .0001
Needs help with ADLs and IADLs^b^				
Needs help with ≥2 ADLs^d^	2.3	15.8	0.8	*p* < .0001
Needs help with ≥2 IADLs^e^	6.2	38.4	0.8	*p* < .0001
Weight risk				
Underweight (BMI < 18.5)^b^	2.0	2.5	1.9	*p* < .05
Obese (BMI ≥ 30)	24.3	32.7	22.8	*p* < .0001
Low exercise frequency (< Once a week or never)	12.2	30.2	9.3	*p* < .0001
No dental exam or teeth cleaning in past 12 months	19.4	31.6	17.5	*p* < .0001
Perception of how well can take care of self				
Very well/completely able	85.8	44.5	92.6	*p* < .0001
Fairly well	11.0	34.9	7.0	
Not very well/not at all able	3.2	20.6	0.4	*p* < .0001

Estimates are based on self-reported survey data weighted to the age-sex composition of KPNC members in 2014

^a^Seniors were considered frail if the average of their frailty point score was ≥ 0.2 based on dividing total frailty points by the 34 point maximum

^b^Characteristic used as part of the FI calculation

^c^Chronic diseases included: diabetes, hypertension, history of heart disease, history of stroke, history of cancer (other than just skin cancer), COPD/chronic bronchitis, Parkinson’s disease, osteoporosis, and osteoarthritis

^d^ADLs include: bathing, dressing, eating, getting in and out of bed or chairs, using the toilet

^e^IADLs include: preparing meals, doing laundry/chores, shopping, managing money, taking medication, using the phone

Mobility limitations and frequent problems with balance or walking were significantly more common among the frail group compared to the non-frail group, and frail adults were significantly more likely to have had ≥1 fall (56.1% vs. 19.7%, p < .0001) and ≥ 2 falls (33.3% vs. 7.7%, *p* < .0001) in the prior 12 months. More than twice the percentage of frail older adults compared to non-frail older adults regularly took medications that can increase the risk of falling, including anti-depressants, anti-anxiety medications, pain medications, and prescription or over-the-counter sleep medicine. Frail older adults were also more likely to have vision problems that affected their daily activities, hearing problems, and oral health problems that affected eating or speech, and they were less likely to be getting preventive dental care. While extremely low percentages of frail and non-frail older adults were underweight, the prevalence of obesity was significantly higher in the frail group (32.7% vs 22.8%, *p* < .001), likely due in part to the higher prevalence of sedentary lifestyle (exercise < once a week).

While 44.5% of the frail group felt that they could take care of themselves very well or completely, approximately 20.6% felt that they could not take care of themselves at all or not very well. In terms of the type of help needed with activities of daily living, 21.7% needed help with bathing, 18.5% with transferring from sitting or prone positions, 12.7% with dressing, 6.2% with using the toilet, and 3.2% with eating. Higher percentages needed help with instrumental activities of daily living, including cutting their toe nails (51%), getting to places out of walking distance (39.9%), doing routine household chores (40.3%), shopping for groceries (37.9%), preparing meals (28.3%), doing laundry (28.1%), managing and taking medicines (17.3%), managing money (13.5%), and using the phone (8.6%). Frail women were significantly more likely than frail men to report needing help getting to places they could not walk to (44.0% vs. 33.3%, *p* < .05), with routine chores (46.9% vs. 29.9%, *p* < .01), and shopping (43.7% vs. 28.6%, *p* < .01). With the exception of being underweight, significant differences between the frail and non-frail groups persisted after we compared age-standardized prevalence estimates and used logistic regression models to control for age and sex.

### Frailty and social determinants of health

Table 4 shows that frail older adults were significantly more likely than non-frail adults to have a low level of educational attainment and low household income, and this difference persisted after adjusting for age. When household income was stratified by sex, frail women were more than twice as likely as frail men to have a household income of ≤ $25,000 (44.0% vs. 17.4%, *p* < .0001). Frail older adults were twice as likely as non-frail to worry a great deal about their financial situation (26.9% vs. 12.6%, *p* < .001). They were also more likely to say that due to cost, in the prior 12 months they had used less medication than prescribed (7.4% vs. 3.6%, *p* < .001), received less medical care than they thought they needed (8.3% vs. 3.7%, *p* < .001), and eaten less fruits and vegetables than they would have (9.5% vs. 3.2%, *p* < .001).

**Table 4:** Social determinants of health by frailty status

	All	Frailty Status		
	(*N* = 4551) Wtd.%	Frail^a^ (*N* = 647) Wtd. %	Non-Frail (*N* = 3904) Wtd. %	*p*-value Frail vs. Non-Frail
Low educational attainment				
≤ High school graduate	24.7	36.6	22.8	*p* < .0001
< High school graduate	4.3	10.0	3.3	*p* < .0001
Household income/financial strain				
≤ $35,000 in past year^b^	26.6	51.4	22.5	*p* < .0001
≤ $25,000 in past year^b^	14.8	33.6	11.7	*p* < .0001
Worries a great deal about financial situation	14.6	26.9	12.6	*p* < .0001
During the past 12 months, due to cost:				
Used less medication than prescribed	4.2	7.4	3.6	*p* < .0001
Got less medical care than thought needed	4.4	8.3	3.7	*p* < .0001
Ate less fruits and vegetables than would have	4.1	9.5	3.2	*p* < .0001
Social circumstances				
Not married or in a committed relationship	33.7	46.6	31.5	*p* < .0001
Has someone can call for help or to arrange for help if needed	94.6	93.1	94.9	ns
Often feels lonely or socially isolated	4.0	13.1	2.5	*p* < .0001
Feels depressed or sad much for most of the time	3.2	9.1	2.3	*p* < .0001
Feels dissatisfied with life	6.9	22.4	4.3	*p* < .0001
Serves as unpaid caregiver to a relative or friend with a serious illness or physical/mental disability	25.5	19.8	26.4	*p* < .01
Does not use the internet or email at all or needs someone to help or use the internet/email for them	24.8	51.6	22.3	*p* < .0001

Estimates are based on self-reported survey data weighted to the age-sex composition of KPNC members in 2014

^a^Seniors were considered frail if the average of their frailty point score was ≥0.2 based on dividing total frailty points by the 34-point maximum

^b^Based on the 92% of respondents for whom income data were available

With regards to social circumstances, nearly half the frail group (46.6%) were not married or in a committed relationship, with more than twice as many frail women not in a committed relationship as compared to men (80.7% vs. 36.4%, *p* < .0001). About 13% of frail adults often felt lonely or socially isolated, 9% felt depressed much of the time, and over 20% expressed dissatisfaction with their life. Over half (51.6%) did not use internet or email on their own. About 92% of frail older adults indicated they had at least one person they could call on for help or to arrange for help if needed, but who they would rely on differed by sex. Men were more likely to indicate that they would count on their spouse for help (79.5% vs. 34.6%, *p* < .0001), while women were more likely to indicate that they would count on a relative (69.6% vs. 29.9%, *p* < .0001), with approximately equal percentages indicating a friend or other non-relative (11.7% of women and 6.6% of men, *p* < .06). Despite being frail themselves, nearly 20% of frail adults provided unpaid caregiver support to an adult who was frail or had a serious illness or disability, with women more likely to have this role than men (22.7% vs. 15.3%, *p* < .05). These significant differences persisted even after adjusting for age.

### Potential single-item screener for frailty

We examined the performance of the single question, “Considering all things, how well can you take care of yourself at this time,” a survey response not used in the FI, as a predictor of frailty in our study cohort. Using unweighted data, we found that 132/148 (89.2%, CI: 84.2–94.2%) of those who reported that they could not take care of themselves at all or not very well, 207/495 (41.8%, CI: 37.5–46.2%) of those who said they could take care of themselves “fairly well”, and 302/3573 (7.8%, CI: 7.0–8.6%) of those who said that could take care of themselves “very well” or “completely able” were classified as frail. Use of the combined response categories “Not at all/not very well” vs. ≥ Very well to predict frailty had a sensitivity of 20.6% (CI: 17.6–24.0%), specificity of 99.6% (CI: 99.3–99.8%), positive predictive value of 89.2% (CI: 82.8–93.4%), and negative predictive value of 88.4% (87.3–89.2%). Using the response category “Fairly well” vs. ≥ Very well to predict frailty had a sensitivity of 40.7% (CI: 36.4–45.1%), specificity of 92.5% (CI: 91.7–93.3%), positive predictive value of 41.8% (CI: 37.5–46.3%), and negative predictive value of 92.2% (91.3–93.0%).

## Discussion

In this large cross-sectional study of community-dwelling adults ages 65 to 90 years who were members of a Northern California health plan with an integrated healthcare delivery system model, we examined the prevalence of frailty and identified potentially modifiable and non-modifiable health risks and social-economic determinants of health that healthcare providers can utilize to intervene and request additional geriatrician support. Using a deficits accumulation model created in line with previous guidelines, we found an overall prevalence of frailty of 14.3% with frailty increasing for both men and women starting in the 75–79 year-old group and trending toward greater prevalence among women as compared with men at higher ages. Consistent with prior studies, frailty was also more common among older adults with low levels of education and income [4, 7–9]. In our study cohort, the prevalence of frailty did not differ by race/ethnicity.

The prevalence of frailty described by prior studies varied widely, ranging from 5 to 58% in one review article [18] to 4% to 59.1% in another review article [10]. This wide range highlights the lack of consensus on how to define and operationalize frailty. The prevalence of frailty calculated in this study population was lower than the 22.7% calculated in a previous study that also used a deficits accumulation model [9]. Factors that may have contributed to the lower prevalence in our study included exclusion of participants over 90 years of age and the fact that our findings were based on self-reported data obtained from a survey of community-dwelling older adults, thus excluding adults with dementia or in skilled nursing facilities who are more likely to be frail [19]. Additionally, care management programs for patients with diabetes mellitus, congestive heart failure and asthma, as well as cardiovascular risk-reduction programs using cardioprotective medications, may have resulted in an overall improved health status of KPNC senior members [20]. The significant increase in prevalence of frailty starting at age 75 reflects findings from other studies demonstrating that the burden of frailty is more prominent among the older aging population [21]. Prior studies have shown that older adults are not a homogenous group [22, 23], with the frail group contributing to higher medical costs even after controlling for comorbidities [24]. This highlights the need to create patient-centered screening and interventions for frail adults and older adults at elevated risk of becoming frail.

With the shortage of geriatricians, care management, including screening and interventions, for frail older adults will increasingly become the responsibility of adult primary care physicians (PCPs). Although there is no gold standard for frailty measurement in the primary care setting, prior studies have shown screening tools useful to direct appropriate interventions [25, 26]. As PCPs already screen for a multitude of chronic conditions within a limited amount of time [27], efforts should be utilized to improve screening tools with an additional focus on training ancillary and administrative staff members to use these tools to target the appropriate older adult populations. A single screening question would help alleviate some of these constraints. Our findings support the need for future studies to determine whether a patient’s perception of how well they can take care of themselves identifies older adults with very low, moderate, or very high likelihood of being frail. The latter two groups, which in our study comprised about half the cohort, could then be further assessed for specific frailty characteristics and risks. Most health-related risks found in this study have potential interventions. About two-thirds of our frail group had frequent or ongoing pain, which likely interfered with their daily activities and participation in exercise. Prior studies have shown physical training, including resistance training, may provide some benefit in preventing frailty, but that exercise alone may not improve functioning [28, 29]. A multidisciplinary approach including behavioral changes, nutritional interventions, and cognition exercises coupled with exercise could be the interventions needed to prevent further development of frailty [29]. Over a third of our frail individuals had problems with insomnia (falling or staying asleep), about 11% are “short sleepers” (≤5 h/day), and about 15% are “long sleepers” (> 9 h/day). Prior studies have shown associations with worsening sleep quality and increasing frailty [30], with the primary causes being treatable conditions such as insomnia and sleep apnea [31, 32]. Insomnia and “short sleep” have also been shown to increase risk of falls among community-dwelling older adults [33–35], and “short sleep” with an increased risk of cognitive impairment [36]. As expected, our frail group had a higher prevalence of falls compared to the non-frail group, but also a higher prevalence of use of medications that increase the risk of falling. Frail patients who are chronically using these types of medications or having problems for which these medications might be prescribed could potentially be tried on non-pharmacological approaches to managing insomnia, pain, depression, and anxiety, such as cognitive behavioral therapy, mindfulness meditation, yoga or gentle movement therapies, and exercise.

Dentition and other oral health problems should be a priority for frail older adults because these can lead to poor oral intake and involuntary weight loss [37], as well as systemic inflammation from gum disease that can increase risk of cardiovascular disease [38]. We found that over 30% of frail adults in our cohort had not had a dental check-up in the past 12 months, suggesting that oral health problems are likely to go undetected unless an examination of the mouth, teeth, and gums becomes part of the annual primary care physical examination for people who do not report having seen a dentist within the prior year.

Counterintuitive to the thought that frailty is a wasting disorder [4] but consistent with prior studies [39], we found a higher prevalence of obesity among the frail group, which could be explained by the obese population having limited mobility (or conversely that limited mobility lead to an obese state), requiring more assistance with ADLs/IADLs and having more chronic conditions [40], all factors included in our FI. Many frail individuals were also found to require more assistance with ADLs/IADLs including household chores, grocery shopping, preparing meals, and doing their laundry, which may highlight the need for continual guidance in regard to community resources and commercial programs that provide these services rather than depending on family and friends alone.

The social determinants of health analyzed in this study may or may not be modifiable among the frail population but are important to take into account in care planning. In line with prior studies [4, 6, 8, 9], the prevalence of frailty was highest among lower education levels, which likely represents a lower health literacy that would contribute to the poor lifestyle choices seen in frailty [41]. Financial strain and lower income level can impact seeking of needed medical and dental care, adherence to medical and medication regimens, and engagement in healthy self-care practices such as eating ≥5 servings of fruits and vegetables per day. Community services, such as home meal delivery programs to address the need for healthier diets, are available in certain areas, though the limitations of cost, availability of the programs, and sparse availability of research regarding outcomes with these programs still remain a concern [42]. Prior studies have also shown that hearing problems and frailty are associated with social isolation and depression [43]. As such, PCPs will increasingly need to involve instrumental social support systems of the patients including unpaid caregivers, social workers, or patient navigators to help provide necessary resources for the patients and families. Interestingly, nearly three-fourths of male seniors who are frail would depend on their spouses for help if they got sick or injured or needed special care. Assuming that the spouses are of similar age, that puts a great caregiving burden on female spouses who may also be dealing with limitations of their own.

This study has some limitations, including the cross-sectional study design and exclusion of non-English speaking members (the MHS questionnaire is only available in English), those living in nursing care or other institutional facilities, and those with cognitive deficits or severe dementia. Other studies have shown that ethnic minorities including Hispanic populations, and individuals in residential settings have a higher prevalence of frailty [22, 44]. Furthermore, this study was unable to validate the frailty index using participants' health records, but did follow the guidelines suggested by Searle et al. [8] for establishing a frailty index and chose deficits based on prior validated frailty indices including the Canadian Study of Health and Aging 70-item Frailty Index [6] and the Frailty Indices used by Searle et al. [8] and Song et al. [9]. There is also possible incorporation bias for the single-item screening question given that mobility is a component of the frailty index; however, future studies can compare this single question to other frailty indices for further examination. One of the strengths of this study is the large number of culturally diverse participants, which provides a more stable estimate of the prevalence and characterization of the frail subgroup. The frail cohort found in this study had similar demographic characteristics to all survey respondents aged 65–90 in regarding to mean age, sex, relationship status, level of education, income status, number of chronic medical conditions, self-perception of physical health, and history of falls in the past 12 months. Importantly, this study characterizes modifiable and non-modifiable risk factors in a contemporary and community-dwelling population of older adults, where those at highest risk for adverse health outcomes and subsequent utilization of healthcare resources can be further identified in clinical practice.

## Conclusions

The frail older adult population is heterogeneous and thus frail patients and those at risk for becoming frail require a patient-centered assessment of their circumstances by primary care providers to try to improve their quality of life, avoid adverse health events such as falls, and slow physical and mental decline. The same set of characteristics identified in this study can proactively be used to assess all older adults to improve primary prevention of frailty and patient health and quality of life.

## Abbreviations

ADL: Activities of Daily Living
BMI: Body Mass Index
FI: Frailty Index
IADL: Instrumental Activities of Daily Living
KPNC: Kaiser Permanente Northern California
MHS: Member Health Survey
PCPs: Primary Care Physicians
SDOH: Social Determinants of Health
Wtd.: Weighted
